# Bias from questionnaire invitation and response in COVID-19 research: an example using ALSPAC

**DOI:** 10.12688/wellcomeopenres.17041.2

**Published:** 2022-07-08

**Authors:** Alba Fernández-Sanlés, Daniel Smith, Gemma L Clayton, Kate Northstone, Alice R Carter, Louise AC Millard, Maria Carolina Borges, Nicholas John Timpson, Kate Tilling, Gareth J Griffith, Deborah A Lawlor

**Affiliations:** 1 MRC Integrative Epidemiology Unit, University of Bristol, Bristol, UK; 2 Population Health Sciences, Bristol Medical School, University of Bristol, Bristol, BS8 2BN, UK; 3 Bristol National Institute of Health Research (NIHR) Biomedical Research Centre, Bristol, UK

**Keywords:** COVID-19, COVID-19 risk factors, Selection Bias, Longitudinal Study, ALSPAC, Missing Data, Questionnaire Invitation, Questionnaire Response.

## Abstract

**Background:** Longitudinal studies are crucial for identifying potential risk factors for infection with, and consequences of, COVID-19, but relationships can be biased if they are associated with invitation and response to data collection. We describe factors relating to questionnaire invitation and response in COVID-19 questionnaire data collection in a multigenerational birth cohort (the Avon Longitudinal Study of Parents and Children, ALSPAC).

**Methods:** We analysed online questionnaires completed between the beginning of the pandemic and easing of the first UK lockdown by participants with valid email addresses who had not actively disengaged from the study. We assessed associations of pre-pandemic sociodemographic, behavioural, anthropometric and health-related factors with: i) being sent a questionnaire; ii) returning a questionnaire; and iii) item response (for specific questions). Analyses were conducted in three cohorts: the index children born in the early 1990s (now young adults; 41 variables assessed), their mothers (35 variables) and the mothers’ partners (27 variables).

**Results:** Of 14,849 young adults, 41% were sent a questionnaire, of whom 57% returned one. Item response was >95%. In this cohort, 78% of factors were associated with being sent a questionnaire, 56% with returning one, and, as an example of item response, 20% with keyworker status response. For instance, children from mothers educated to degree-level had greater odds of being sent a questionnaire (OR=5.59; 95% CI=4.87-6.41), returning one (OR=1.60; 95% CI=1.31-1.95), and responding to items (e.g., keyworker status OR=1.65; 95% CI=0.88-3.04), relative to children from mothers with fewer qualifications. Invitation and response rates and associations were similar in all cohorts.

**Conclusions:** These results highlight the importance of considering potential biases due to non-response when using longitudinal studies in COVID-19 research and interpreting results. We recommend researchers report response rates and factors associated with invitation and response in all COVID-19 observational research studies, which can inform sensitivity analyses.

## Introduction

Severe acute respiratory syndrome coronavirus-2 (SARS-CoV-2) has infected more than 181 million individuals worldwide and is responsible for over 3.9 million global deaths to date (1 ^st^ July 2021, WHO). Due to their wealth of pre-pandemic data, prospective longitudinal studies are making important contributions to understanding the mechanisms of both infection and disease (COVID-19), and the impact of the pandemic and its management on future health ^1– 6^. However, sociodemographic, behavioural and health-related factors may shape not only who gets infected, progression to COVID-19 and disease severity, but also health-seeking behaviour, who gets tested, and their wider response to the pandemic (e.g., mental health impact). Furthermore, these factors may be associated with, or even influence, who is assessed or sent a questionnaire, and who responds to questionnaires or items they contain. This can result in selection bias ^7– 11^, which may be exacerbated by the ways in which data collection has changed during the pandemic, for example, with the increased use of online questionnaires ^12, 13^. This selection bias can lead to incorrect (or biased) estimations of the effect of a risk factor on an outcome, in this case COVID-19 and its related impacts. Importantly, having an available and rich collection of pre-pandemic data in existing longitudinal cohorts can be useful in efforts to explore potential selection pressures that lead to bias ^14– 17^.

The aim of this study was to describe questionnaire invitation and response rates and to explore factors associated with (i) being sent a COVID-19 questionnaire (i.e., participants who were invited to complete a questionnaire because they had not withdrawn from the study, agreed to participate in questionnaires and had a valid email address); (ii) returning a COVID-19 questionnaire; and (iii) item response (for six key variables: self-reported COVID-19 status, predicted COVID-19 cases based on symptoms ^18^, three mental health outcomes [depression, anxiety and well-being] and keyworker status), in the Avon Longitudinal Study of Parents And Children (ALSPAC), a multi-generational longitudinal study based in the South West of England established in the early 1990s. Throughout this paper we use ‘being sent a questionnaire’ and ‘questionnaire invitation’ synonymously, and include both returning a questionnaire and item completion as ‘response’. We focused on the three adult cohorts over two generations: the index participants born in the early 1990s, Generation-1 (G1); their mothers, Generation-0 (G0) mothers; and the mothers’ partners (G0 partners). For each outcome, we examined multiple candidate predictors of invitation and response encompassing a range of sociodemographic, behaviour, anthropometric and health-related factors. We focused our analyses on the first two online COVID-19 questionnaires, the first completed between 9 ^th^ April and 15 ^th^ May 2020, and the second between 26 ^th^ May and 5 ^th^ July 2020 ^19, 20^.

## Methods

### Study design

ALSPAC is a three-generation birth cohort that started recruiting pregnant women resident in the former county of Avon (centred around the city of Bristol, UK), with delivery dates between April 1991 and December 1992. A total of 14,541 pregnancies were initially enrolled (14,676 foetuses), resulting in 13,988 children alive at one year of age. Those women (G0 mothers), their partners (G0 partners) and their index children (G1) have been followed with regular assessments since this time. Since the oldest children were approximately 7 years of age, the study has recruited 913 additional G1 children who did not join originally, but were part of the original target population based on date and location of birth. Hereafter we will refer to the participants where the G1 index child was alive at one year of age and who did not withdraw consent for their data to be used as the “whole cohort” (14,849 G1 children; 14,282 G0 mothers; 14,275 G0 partners). The target population therefore comprises pregnancies leading to children born in the early 1990s in the former county of Avon, in addition to their mothers and the mothers’ partners. This initial enrolled sample included approximately 75% of the target population and was broadly representative of the wider Avon population (albeit somewhat biased towards mothers who were married and from higher socioeconomic backgrounds, and biased away from ethnic minorities); further details can be found in the published cohort profiles ^14, 21, 22^. The study website contains details of all the data available through a fully searchable data dictionary and variable search tool.

Since the start of the pandemic, participants have been sent four online COVID-19 questionnaires to assess diagnoses, symptoms, and behavioural and environmental factors related to COVID-19, and the impact of the pandemic on health ^19, 20, 23, 24^. Questionnaires were sent to all participants who had not withdrawn from the study or declined to participate in questionnaires, and had a valid email address in the ALSPAC administrative records. Some participants would have provided updated email address information or re-engaged with the study between the questionnaires, while other participants may have withdrawn from the study during this time. Therefore, the number of participants invited to these questionnaires may differ. Data for the COVID-19 questionnaires were collected and managed using REDCap electronic data capture tools hosted at the University of Bristol ^25^. In this study we focused on the first two questionnaires ^19, 20^. Analyses were conducted for each COVID-19 questionnaire separately (COVIDQ1 and COVIDQ2) and combined (data from both COVIDQ1 *and* COVIDQ2, and data from either COVIDQ1 *or* COVIDQ2).

### COVID-19 questionnaire invitation, return and completion

**Figure 1.**
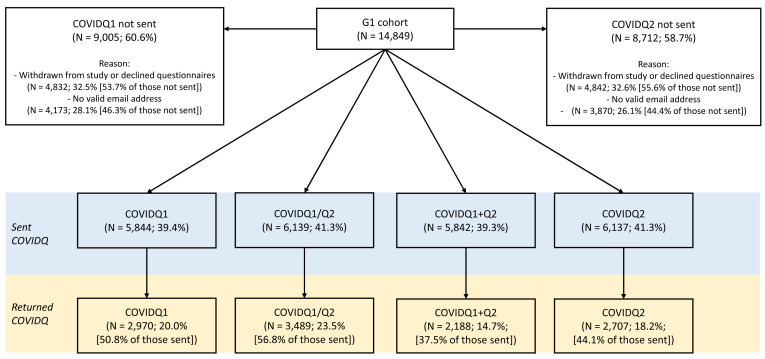
Flowchart of COVID-19 questionnaire invitation and response in the G1 cohort. COVIDQ1 and COVIDQ2 refer to the first and second COVID-19 questionnaires, respectively, COVIDQ1/Q2 refers to being sent/returning either COVIDQ1 or COVIDQ2, and COVIDQ1+Q2 refers to being sent/returning both COVIDQ1 and COVIDQ2. Details of reasons why participants were not sent a COVIDQ1 or COVIDQ2 questionnaire are also given.

**Figure 2.**
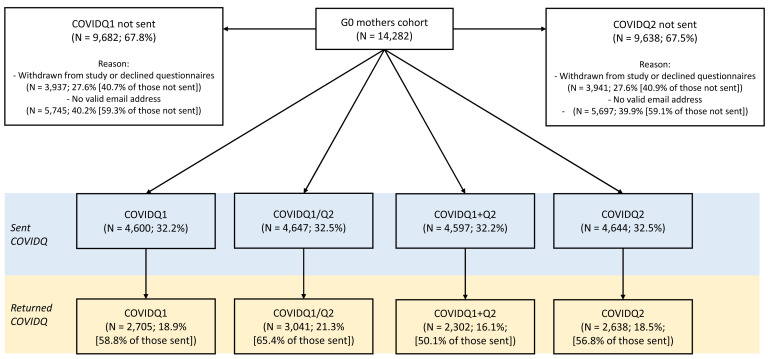
Flowchart of COVID-19 questionnaire invitation and response in the G0 mothers cohort. COVIDQ1 and COVIDQ2 refer to the first and second COVID-19 questionnaires, respectively, COVIDQ1/Q2 refers to being sent/returning either COVIDQ1 or COVIDQ2, and COVIDQ1+Q2 refers to being sent/returning both COVIDQ1 and COVIDQ2. Reasons why participants were not sent a COVIDQ1 or COVIDQ2 questionnaire are also given.

We described invitation and response rates, and examined associations with the following three outcomes ( Table 1; Figure 1 - G1 cohort, Figure 2 - G0 mothers cohort, *Extended data* ^26^: Figure S1 - G0 partners cohort):

**Table 1.** Description of the outcome variables.

Outcome	Description	Sample	Comparison	Variable name (COVID1; COVID2)	Notes
Being sent a COVID-19 questionnaire	Whether the participant was sent a COVID-19 questionnaire	Whole cohort	Sent vs Not sent	covid1_0001; covid2_0001	_
Returning a COVID-19 questionnaire	Whether the participant returned a COVID-19 questionnaire	Participants who were sent a COVID-19 questionnaire	Returned vs Not returned	covid1_0002; covid2_0002	_
Having self-reported COVID-19 data	Whether the participant answered the question on self-reported COVID-19 diagnosis	Participants who returned a COVID-19 questionnaire	Responded vs Missing	covid1_2580; covid2_1060	_
Having predicted COVID-19 data	Whether the participant has data for predicted COVID-19 case, estimated using the Menni algorithm based on responses to a checklist of COVID-19 related symptoms	Participants who returned a COVID-19 questionnaire	Responded vs Missing	covid1_2605; covid2_1030	For participants with missing data for specific symptoms, unless all questions were blank it was assumed that the participant did not experience that symptom.
Having SMFQ data	Whether the participant has SMFQ total score data (depression)	Participants who returned a COVID-19 questionnaire	Responded vs Missing	covid1_4065; covid2_4065	Total score comprised of 13 SMFQ items. Participants missing ≥1 SMFQ item were coded as missing.
Having GAD-7 data	Whether the participant has GAD-7 total score data (anxiety)	Participants who returned a COVID-19 questionnaire	Responded vs Missing	covid1_4080; covid2_4080	Total score comprised of 7 GAD-7 items. Participants missing ≥1 GAD-7 item were coded as missing.
Having WEMWBS data	Whether the participant has WEMWBS total score data (well-being)	Participants who returned a COVID-19 questionnaire	Responded vs Missing	covid1_4120; covid2_4120	Total score comprised of 14 WEMWBS items. Participants missing ≥1 WEMWBS item were coded as missing.
Having keyworker status data	Whether the participant answered the question about being a keyworker	Participants who returned a COVID-19 questionnaire	Responded vs Missing	covid1_5045; covid2_6030	_

SMFQ, Short Mood and Feelings Questionnaire; GAD-7, Generalised Anxiety Disorder seven-item Assessment; WEMWBS, Warwick-Edinburgh Mental Wellbeing Scale.

**   1.   Outcome: Being sent a COVID-19 questionnaire**
Sample: All enrolled participants for which the G1 child was alive at 1 year old and who had not withdrawn consent for their data to be used.
Reference group: Participants who were not sent a COVID-19 questionnaire because they had withdrawn from the study, declined to participate in questionnaires or did not have a valid email address.
**   2.   Outcome: Returning a COVID-19 questionnaire**
Sample: Participants who were sent a COVID-19 questionnaire.
Reference group: Participants who, having been sent a COVID-19 questionnaire, did not return it.
**   3.   Outcome: Completing items that define six key variables which may be relevant to wider COVID-19 research (item response)**
Sample: Participants who returned a COVID-19 questionnaire.
Reference group: Participants who returned the questionnaire but did not provide data on the relevant questionnaire items that define the key variable.

The variables examined are listed below. The original ALSPAC variable names, along with additional details, are provided in Table 1 (see the Wellcome Open Research Data notes of COVIDQ1 and COVIDQ2 for full information on the corresponding questions and how the variables were derived ^19, 20^).

*   a.   Self-reported COVID-19 diagnosis.*
*   b.   COVID-19 case prediction using the Menni algorithm* ^18^, *based on self-reported symptoms.*
*   c.   Total score on the Short Mood and Feelings Questionnaire (SMFQ)* ^27^ *to assess depression.*
*   d.   Total score on the Generalised Anxiety Disorder seven-item Assessment (GAD-7)* ^28^ *to assess anxiety.*
*   e.   Total score on the Warwick-Edinburgh Mental Wellbeing Scale (WEMWBS)* ^29^ *to assess well-being.*
*   f.   Self-reported keyworker status.*

### Candidate predictors of selection

We examined associations of pre-pandemic characteristics (41, 35 and 27 variables for the G1, G0 mother and G0 partner cohorts, respectively; *Extended data* ^26^: Tables S1 and S2) with the pre-specified outcomes defined above. Hereafter we will refer to these as “candidate predictors of selection”, acknowledging that we are not studying all possible sources of selection bias in ALSPAC and its COVID-19 data. We selected those variables *a priori* based on our knowledge of ALSPAC, COVID-19 and factors that are hypothesised to, or are known to, shape patterns of invitation and response ^14, 16, 21, 30^. For analyses involving continuous variables, these were transformed into standard deviation (SD) units in order for all odds ratios to be interpretable on the same scale.

### Statistical analysis

We used unadjusted logistic regression to quantify associations between each candidate predictor of selection and the outcomes detailed above. As we were interested in raw associations between variables, rather than estimating potential causal relationships, we did not perform multivariable analyses to adjust for potential confounders. We did not analyse any outcome with 10 or fewer participants in the reference or sent/returned/item response group (depending on the outcome being assessed). Note that, to aid interpretability of the figures displaying these results, we present a range of key candidate predictors of selection in the main text, with additional predictors displayed in the extended data. All analyses were conducted using R version 4.0.3 ^31^.

To describe the results in the main text, we established an arbitrary criterion based on *p*-values of the associations using a threshold of 0.05. This threshold has been used in similar studies to summarise large numbers of associations ^15^. Weaker, but still potentially relevant, associations may be overlooked using this criterion, so we further described associations where the absolute *z*-value (log point estimate divided by the log standard error) was greater than 1 (equivalent to a *p*-value <0.32). As these thresholds are arbitrary and *p*-values (or *z*-values) do not inform about the magnitude of the association ^32^, we recommend readers consider the magnitude, direction and uncertainty of each association when interpreting these results and when undertaking COVID-19 research using ALSPAC data.

### Ethical considerations

Ethical approval for the study was obtained from the ALSPAC Ethics and Law Committee and the Local Research Ethics Committees. Informed consent for the use of data collected via questionnaires and clinics was obtained from participants following the recommendations of the ALSPAC Ethics and Law Committee at the time. Study participants have the right to withdraw their consent for elements of the study or from the study entirely at any time. Full details of the ALSPAC consent procedures are available on the study website.

## Results

The results of the combined questionnaires are presented in the main text, while the separate COVIDQ1 and COVIDQ2 results are presented in the *Extended data* ^26^. Results for the G0 partners are also presented in the *Extended data* ^26^, as partners of the G0 mothers may change over time, the amount of data for G0 partners is lower than for G1 and G0 mothers (meaning that estimation in this cohort will be less precise), and the G0 partners’ data are used less frequently than the G1 and G0 mothers’ data. Figure S2 ( *Extended data* ^26^) shows the overlap among those who replied to either or both questionnaires, for each cohort.

### Candidate predictors of selection and invitation/response outcomes in the G1 cohort – Combined COVID-19 questionnaires

Amongst the G1 participants, 41% were sent either COVID-19 questionnaire (with 39% sent both), of whom 57% returned at least one (37% returned both). Of those not invited to complete a questionnaire, ~55% had previously withdrawn from the study or declined to receive questionnaires, while ~45% had not actively disengaged but did not have a valid email address. Key variable response was >95% of those who returned either questionnaire for all of the six key variables, with fewer than five participants missing data on self-reported COVID-19 status and predicted COVID-19 status based on symptoms. G1 participant numbers for questionnaire invitation, return and completion are shown in Table 2 and Figure 1.

**Table 2.** Descriptive statistics of the outcomes from the combined COVID-19 questionnaires for the G1 cohort.

Outcome	n	Yes (%)	No (%)
**Sent either questionnaire ^a^**	14,849	6,139 (41.34%)	8,710 (58.66%)
**Sent both questionnaires ^a^**	14,849	5,842 (39.34%)	9,007 (60.66%)
**Returned either questionnaire**	6,139	3,489 (56.83%)	2,650 (43.17%)
**Returned both questionnaires**	5,842	2,188 (37.45%)	3,654 (62.55%)
**Having self-reported COVID-19 status data ^b^**	3,489	>3,484 (>99.86%)	<5 ^c^ (<0.14%)
**Having predicted COVID-19 status (from symptoms; Menni algorithm) data ^b^**	3,489	>3,484 (>99.86%)	<5 ^c^ (<0.14%)
**Having SMFQ total score (depression) data ^b^**	3,489	3,332 (95.5%)	157 (4.5%)
**Having GAD-7 total score (anxiety) data ^b^**	3,489	3,349 (95.99%)	140 (4.01%)
**Having WEMWBS total score (well-being) data ^b^**	3,489	3,330 (95.44%)	159 (4.56%)
**Having keyworker status data ^b^**	3,489	3,343 (95.82%)	146 (4.18%)

SMFQ, Short Mood and Feelings Questionnaire; GAD-7, Generalised Anxiety Disorder seven-item Assessment; WEMWBS, Warwick-Edinburgh Mental Wellbeing Scale. ^a^ Sample based on all enrolled G1 participants where G1 child was alive at one year of age and had not withdrawn consent for their data to be used. ^b^ These item/variable response outcomes are based on whether the participant returned either COVID-19 questionnaire (i.e., having data in either questionnaire). ^c^ Actual numbers withheld due to small cell counts (<5).

**Figure 3.**
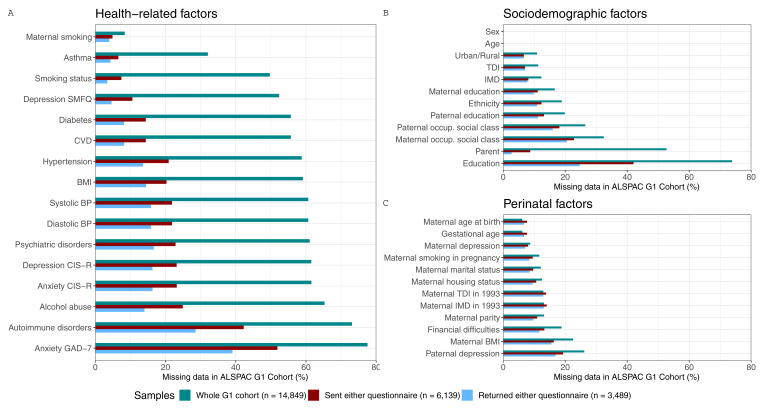
Proportion of missing data in each candidate predictor of selection for the three samples of the G1 cohort. These samples are the whole ALSPAC G1 cohort (green), the subsample who was sent either COVID-19 questionnaire (red), and the subsample who returned either COVID-19 questionnaire (blue). To facilitate the interpretation of the results, variables of related categories are presented in the same panel ( **a**.- health-related variables, which include comorbidities, and behavioural and anthropometric factors; **b**.- sociodemographic factors; and **c**.- perinatal factors) and are ordered by the total amount of missing data in the whole sample. SMFQ, Short Mood and Feelings Questionnaire; CVD, Cardiovascular Disease; BMI, Body Mass Index; BP, Blood Pressure; CIS-R, Clinical Interview Schedule – Revised; GAD-7; Generalised Anxiety Disorder seven-item assessment; IMD, Index of Multiple Deprivation; TDI, Townsend Deprivation Index; occup., occupational.

In the whole G1 cohort, the proportion of missing data for the candidate predictors of selection ranged from no missingness (age and sex) to three-quarters of data missing for some of the more recently collected data (e.g., education). In the G1 sample who were sent either questionnaire, completeness was generally higher, with most variables having less than 25% missingness. In the G1 sample that returned either questionnaire, completeness was higher still, with most individual variables missing less than 17% data. Figure 3 shows the proportion of missing data for each candidate predictor of selection for the three G1 samples. However, when multiple candidate predictors of selection are considered jointly, the sample size will naturally reduce further (e.g., in the whole G1 cohort, 66% of participants would be excluded from models adjusting for recent body mass index (BMI), recent smoking status, maternal education, maternal age and maternal parity; while in the ‘sent either questionnaire’ and ‘returned either questionnaire’ samples the percentage of excluded participants would be 31% and 24%, respectively).

**Figure 4.**
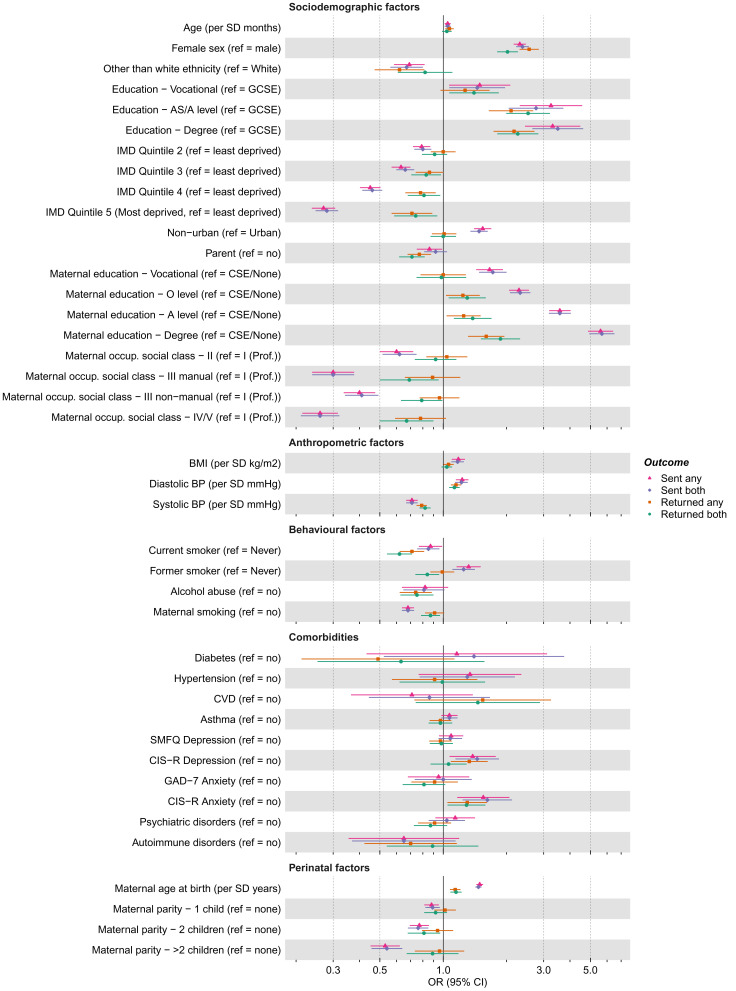
Associations between candidate predictors of selection and being sent and returning either or both of the COVID-19 questionnaires in the G1 cohort. For ease of presentation some candidate predictors of selection are presented in this figure, while others are displayed in Figure S3a. The x-axis is displayed on the logarithmic scale. Means and standard deviations (SD) of continuous variables: Age (months; mean = 337.0, SD = 5.89), BMI (kg/m ^2^; mean = 24.43, SD = 5.05), SBP (mmHg; mean = 117.09, SD = 11.45) and DBP (mean = 65.99, SD = 7.75). Ref, reference; (G)CSE, (General) Certificate of Secondary Education; IMD, Index of Multiple Deprivation; Occup., occupation; Prof., professional; BMI, Body Mass Index; BP, Blood Pressure; CVD, Cardiovascular Disease; SMFQ, Short Mood and Feelings Questionnaire; CIS-R, Clinical Interview Schedule – Revised; GAD-7, Generalised Anxiety Disorder seven-item Assessment.

Differences between the associations of the 41 candidate predictors with being sent or returning either questionnaire (i.e., COVIDQ1 *or* COVIDQ2) and those with being sent or returning both questionnaires (i.e., COVIDQ1 *and* COVIDQ2) were minimal ( Figure 4; *Extended data* ^26^: Figure S3a), so we focus on being sent and returning either questionnaire here. For the purpose of description and at a *p*-value threshold of <0.05, 32 (78%) and 23 (56%) of the 41 candidate predictors were associated with being sent and returning a questionnaire, respectively. Equivalent results using the criteria of an absolute *z*-value ≥1 were 39 (95%) and 32 (78%). Being female, having a higher socioeconomic position, greater BMI, and older maternal age at birth were associated with higher odds of being sent a questionnaire, while ethnicity other than white, higher maternal parity, maternal perinatal depression and mother smoking during pregnancy were associated with lower odds. Similar patterns were seen for associations with returning a questionnaire, but overall effect sizes were smaller.

Figures S3b and S3c ( *Extended data* ^26^) show associations between the 41 candidate predictors of selection with the outcome variables defined by completion of key questionnaire items. We did not analyse those with COVID-19 outcomes (self-reported COVID-19 status and predicted COVID-19 status based on symptoms) as most participants responded to those questions (<5 participants with missing data for these outcomes). Compared to the analyses of being sent and returning a questionnaire, few factors were associated with any of the remaining four outcomes, and results for all four outcomes were similar. For example, of 41 candidate predictors of selection, only 8 (20%) were associated with keyworker item response (at a *p*-value <0.05; 32 [56%] had an absolute *z*-value ≥1). Higher educational attainment and lower maternal age at birth were associated with higher odds of variable response, while current smoking, maternal perinatal depression and being a parent were associated with lower odds.

### Candidate predictors of selection and invitation/response outcomes in the G0 mothers cohort – Combined COVID-19 questionnaire

In total, 33% of G0 mothers were sent at least one COVID-19 questionnaire (32% were sent both), of whom 65% returned at least one (50% returned both; Table 3 and Figure 2). Of those not invited to complete a questionnaire, ~40% had previously withdrawn from the study or declined to receive questionnaires, while ~60% had not actively disengaged but did not have a valid email address. Key variable response was >95% of those who returned either questionnaire for all six key variables, with fewer than five participants missing data on self-reported COVID-19 status and predicted COVID-19 status based on symptoms.

**Table 3.** Descriptive statistics of the outcomes from the combined COVID-19 questionnaires for the G0 mothers cohort.

Outcome	n	Yes (%)	No (%)
**Sent either questionnaire ^a^**	14,282	4,647 (32.54%)	9,635 (67.46%)
**Sent both questionnaires ^a^**	14,282	4,597 (32.19%)	9,685 (67.81%)
**Returned either questionnaire**	4,647	3,041 (65.44%)	1,606 (34.56%)
**Returned both questionnaires**	4,597	2,302 (50.08%)	2,295 (49.92%)
**Having self-reported COVID-19 status data ^b^**	3,041	>3,036 (>99.83%)	<5 ^c^ (<0.17%)
**Having predicted COVID-19 status (from symptoms; Menni algorithm) data ^b^**	3,041	>3,036 (>99.83%)	<5 ^c^ (<0.17%)
**Having SMFQ total score (depression) data ^b^**	3,041	2,914 (95.82%)	127 (4.18%)
**Having GAD-7 total score (anxiety) data ^b^**	3,041	2,944 (96.81%)	97 (3.19%)
**Having WEMWBS total score (well-being) data ^a^**	3,041	2,913 (95.79%)	128 (4.21%)
**Having keyworker status data ^b^**	3,041	2,951 (97.04%)	90 (2.96%)

SMFQ, Short Mood and Feelings Questionnaire; GAD-7, Generalised Anxiety Disorder seven-item Assessment; WEMWBS, Warwick-Edinburgh Mental Wellbeing Scale. ^a^ Sample based on all enrolled G0 mothers where G1 child was alive at one year of age and had not withdrawn consent for their data to be used. ^b^ These item/variable response outcomes are based on whether the participant returned either COVID-19 questionnaire (i.e., having data in either questionnaire). ^c^ Actual numbers withheld due to small cell counts (<5).

**Figure 5.**
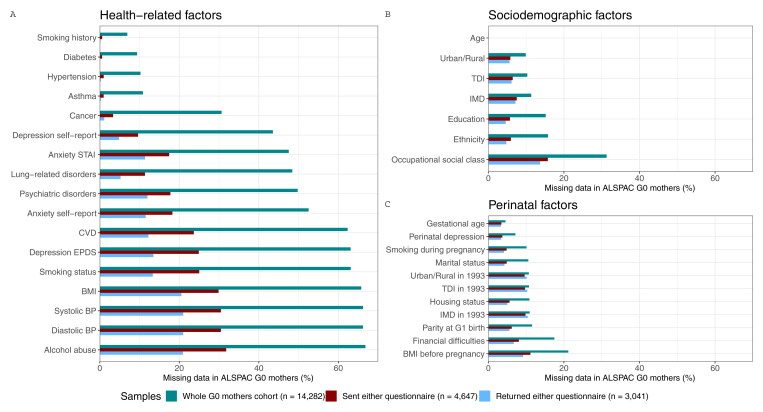
Proportion of missing data in each candidate predictor of selection for the three samples of G0 mothers. These samples are the whole ALSPAC cohort of G0 mothers (green), the subsample who was sent either COVID-19 questionnaire (red), and the subsample who returned either COVID-19 questionnaire (blue). To facilitate the interpretation of the results, variables of related categories are presented in the same panel ( **a**. health-related variables, which include comorbidities, and behavioural and anthropometric factors; **b**. sociodemographic factors; and **c**. perinatal factors) and are ordered by the total amount of missing data in the whole sample comparison. STAI, State-Trait Anxiety Inventory; CVD, Cardiovascular Disease; EPDS, Edinburgh Postnatal Depression Scale; BMI, Body Mass Index; BP, Blood Pressure; IMD, Index of Multiple Deprivation; TDI, Townsend Deprivation Index.

In the whole G0 mothers cohort, the proportion of missing data for the candidate predictors of selection ranged from low/minimal (<20%, for age and most baseline covariates) to two-thirds of data missing for more recently collected data (e.g., alcohol abuse, smoking status, BMI and blood pressure). In the G0 mothers sample that were sent either questionnaire, completeness was generally higher, with all variables having less than 32% missingness. In the G0 mothers sample that returned either questionnaire, completeness was higher still, with most individual variables missing less than 21% data. Figure 5 shows the proportion of missing data in each candidate predictors of selection for the three G0 mothers samples.

**Figure 6.**
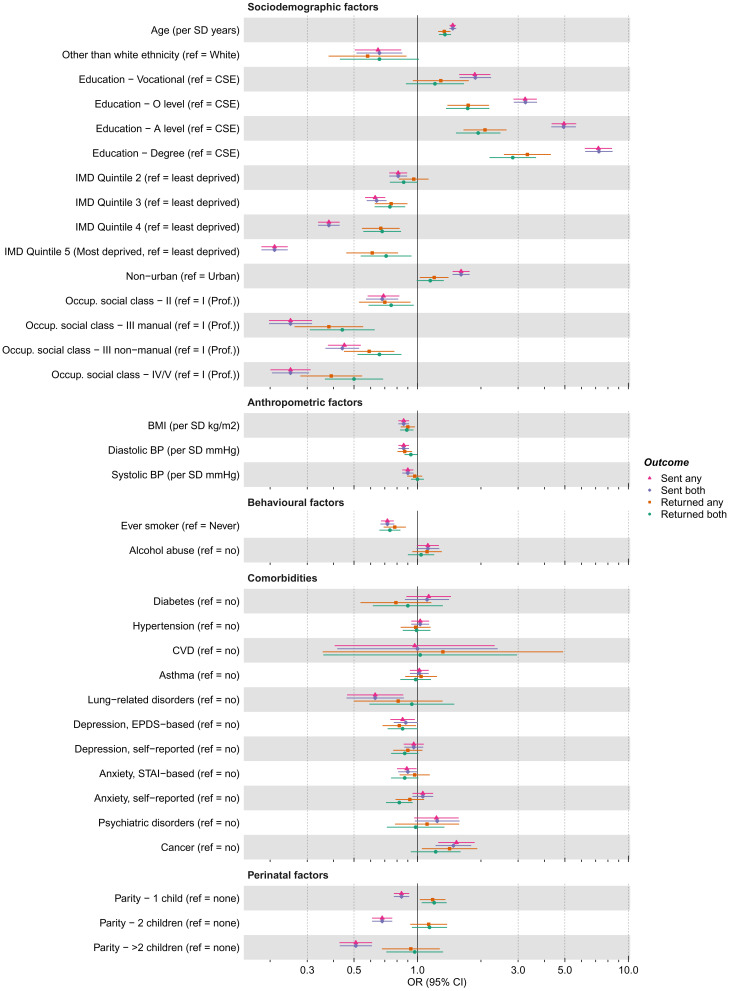
Associations between candidate predictors of selection and being sent and returning either or both of the COVID-19 questionnaires (Q) in the G0 mothers cohort. For ease of presentation some candidate predictors of selection are presented in this figure, while others are displayed in Figure S4a. The x-axis is displayed on the logarithmic scale. Means and standard deviations (SD) of continuous variables: Age (years; mean = 56.14, SD = 5.01), BMI (kg/m ^2^; mean = 26.80, SD = 5.47), SBP (mmHg; mean = 119.93, SD = 14.28) and DBP (mean = 71.36, SD = 9.56). Ref, reference; CSE, Certificate of Secondary Education; IMD, Index of Multiple Deprivation; Occup., occupational; Prof., professional; BMI, Body Mass Index; BP, Blood Pressure; CVD, Cardiovascular Disease; EPDS, Edinburgh Postnatal Depression Scale; STAI, State-Trait Anxiety Inventory.

As with the G1 cohort, differences between the associations of the 35 candidate predictors with being sent or returning either questionnaire and those with being sent or returning both questionnaires were minimal ( Figure 6; *Extended data* ^26^: Figure S4a), so we focus on being sent and returning either questionnaire here. For the purpose of description and at a *p*-value threshold of <0.05, 27 (77%) and 22 (63%) of the 35 candidate predictors were associated with being sent and returning a questionnaire, respectively. Equivalent results using the criteria of an absolute *z*-value ≥1 were 30 (86%) and 26 (74%). Results were broadly similar to the G1 cohort, with factors associated with G0 mothers being sent a questionnaire including: education, area deprivation, occupational social class, older age, White ethnicity, no history of smoking, lower BMI, lower diastolic and systolic blood pressure (DBP and SBP), and several sociodemographic variables measured at baseline (e.g., home ownership status, marital status, parity and financial difficulties). As with the G1 cohort, similar patterns were seen for the associations with returning a questionnaire, but overall effect sizes were smaller.

Figures S4b and S4c ( *Extended data* ^26^) show associations between the 35 candidate predictors with the key variables defined by completion of key questionnaire items. We did not analyse those with COVID-19 outcomes as most participants responded to those questions (<5 participants with missing data for these outcomes). Results were broadly similar to the G1 cohort, with few strong associations between the assessed factors and item response observed, although higher education, lower BMI and no history of smoking were associated with response.

### Candidate predictors of selection and invitation/response outcomes – G0 partners cohort, and first and second COVID-19 questionnaires separately

Compared to the G1 participants and the G0 mothers, a smaller proportion of G0 partners were sent and returned either questionnaire (13% sent either questionnaire, of which 65% returned one; *Extended data* ^26^: Table S3 and Figure S1), and the missingness of the candidate predictors of selection was more substantial ( *Extended data* ^26^: Figure S5). While these results therefore contain greater uncertainty than the G1 and G0 mothers cohorts, there were several candidate predictors of selection associated with being sent and returning a questionnaire among G0 partners, with effect sizes larger for being sent than for returning a questionnaire ( *Extended data* ^26^: Figure S6). Few factors were strongly associated with item response, and effect estimates were somewhat inconsistent with substantial uncertainty ( *Extended data* ^26^: Figure S7). Full details for the G0 partners’ data and results for their analyses using the combined COVID-19 questionnaires are given in the *Extended data* ^26^ (Table S3; Figures S1 and S5–S7).

Results for the first and second COVID-19 questionnaires separately (for G1, G0 mothers and G0 partners) are presented in the *Extended data* ^26^ (Tables S4-S9; Figures S8–S25). They were not materially different to those presented above for the combined datasets. 

## Discussion

Associations between participant characteristics and COVID-19 questionnaire invitation and response matter because they can bias associations of those factors with COVID-19 outcomes. A comprehensive description of the analysed data and relationships with missing data allow informed inferences from association studies; this is possible in longitudinal studies in which missing data can be characterised using previously collected data. In a UK-based multigenerational birth cohort, we have demonstrated that many sociodemographic, behavioural and health-related factors were associated with (i) being sent and (ii) returning COVID-19 questionnaires, and (iii) completion of questions within these questionnaires related to six key variables for COVID-19 research. Generally, the magnitude of associations for being sent a questionnaire were larger than for returning a questionnaire. Of those who returned a questionnaire, there were fewer predictors of item response, but, due to the smaller sample size and generally high response rates, effect sizes were estimated with greater uncertainty than for being sent or returning a questionnaire. Results were similar in all three ALSPAC cohorts (G1, G0 mothers and G0 partners).

Our findings show that – on average – participants who were invited and responded to these COVID-19 questionnaires are different in several key characteristics from those who were not invited, did not return a questionnaire and did not complete all its items. In all cohorts, fewer than half of participants were sent a questionnaire (41% in G1, 33% in G0 mothers and 13% in G0 partners). While questionnaire return rates were relatively high (57% of G1 participants, and 65% of participants in both G0 cohorts, returned either questionnaire) with mostly complete item/variable response (all >95%), together this results in considerable levels of missing data (e.g., for G1 and G0 mothers only ~20% of the whole cohort have COVID-19 questionnaire data; <10% for G0 partners). Recruitment was intended to be representative of the target population; however, due to loss to follow-up, the analytic samples with COVID-19 questionnaire data are no longer representative of the original ALSPAC study sample (and hence the target population, of which the original ALSPAC sample is broadly representative). Selection bias may therefore be a potential risk when using ALSPAC COVID-19 questionnaire data and we encourage researchers to carefully consider the results presented here to inform their work.

Two key points are worth highlighting when interpreting these findings. First, as we were interested in the raw associations between variables, we did not account for any confounding between the candidate predictors of selection, and our findings should not be interpreted as evidence for a certain factor to be independently associated with the outcomes assessed. Second, the impact of missing data needs to be considered carefully as variables from more recent data collections are biased towards certain groups (e.g., higher socioeconomic position [SEP], older G0 mothers, female G1 participants), which may result in selection bias in the observed associations. We also note that there may be selection bias when examining candidate predictors of returning a questionnaire (as this is conditional on being sent a questionnaire) and item response (as this is conditional on being sent and returning a questionnaire) due to unmeasured confounders of questionnaire invitation and response.

To illustrate these two key points, we selected a history of cancer among G0 mothers, which was associated with both being sent and returning a questionnaire. These associations may be due to confounding, as age and SEP both predict questionnaire invitation and response and may also predict cancer diagnosis (since older people are more likely to suffer from cancer and people from higher SEP backgrounds may also be more likely to engage in health-seeking behaviours ^33^ and, therefore, potentially be diagnosed with cancer). When we adjusted for age and education (a proxy for SEP), these associations were somewhat – although not completely – attenuated ( *Extended data* ^26^: Table S10).

Additionally, data on cancer is missing for ~30% of G0 mothers, and it is likely that missing data is associated with characteristics such as age and SEP ( *Extended data* ^26^: Figure S26), which may result in biased associations. We present an example to illustrate this. When the associations of age (a nearly fully-observed predictor) with being sent or receiving either questionnaire were analysed in all participants regardless of having cancer data (an unbiased estimate), or only in those with recorded cancer data, the odds ratio estimates for being sent a questionnaire differed, indicating potential bias ( *Extended data* ^26^: Table S11). Candidate predictors of selection with missing data may therefore result in biased associations with questionnaire invitation and response due to selection bias.

We have not aimed to mitigate selection bias in this paper, but rather to illustrate how it can be identified. Methods such as multiple imputation, inverse probably weighting, *g*-formula approaches, simulations, and bounds and parameter searches ^10, 11, 30, 34– 38^ can be used to help explore and overcome potential selection bias. For instance, in the case of inverse probability weighting, weights should be derived according to the target population of the study (or using the total enrolled ALSPAC sample as a proxy, if weights derived from the target population cannot be constructed). For some examples of such methods applied in ALSPAC to mitigate potential selection bias, see 16, 39– 42. Researchers need to assess the assumptions when using these approaches as it is not possible to dictate a ‘one-size-fits-all’ approach when working with cohort data such as ALSPAC, as different research questions will be addressed using different variables and methods. However, the number of potential predictors of selection and magnitude of their associations with being sent a questionnaire were larger than for questionnaire and item response, suggesting that much – although by no means all – of the potential selection bias reported here could be minimised by using these variables as weights (if using inverse probability weighting), or auxiliary variables (if using multiple imputation) when analysing these data. In addition, as questionnaire invitation could not have been affected by the COVID-19 pandemic – since disengagement from the study and having a valid email address largely occurred prior to the pandemic – selection due to being sent a questionnaire cannot be caused by COVID-19 outcomes. That said, promotion of ALSPAC’s COVID-19 data collections could have prompted some participants to re-engage and provide the study with an up-to-date email address. In the period of interest for this study (between 9 ^th^ April 2020 and 5 ^th^ July 2020), we estimate that this may have been the case for a few hundred participants.

This descriptive study also demonstrates that longitudinal studies allow researchers to utilise their rich detailed pre-pandemic data as potential predictors of selection in COVID-19 studies that inform their research. While some characteristics may have a similar impact on selection across various studies (such as SEP predicting selection, as found in ALSPAC and the 1958 British birth cohort ^17^), others are likely to be study-specific. For instance, in the ALSPAC G1 cohort, participants enrolled during their mother’s pregnancy or as children, while in other cohorts participants may have enrolled as adults (e.g. UK Biobank). We therefore cannot assume that the results described here will apply to other studies with different demographic profiles, enrolment strategies and data collection mechanisms. Additionally, given the target population (defined above), ALSPAC is not necessarily representative of the general UK population or non-UK populations, as: i) at time of recruitment, the Bristol area comprised mainly White Europeans; and ii) the cohort that includes both females and males is a young population (G1, aged ~28 years old), while the sex-specific G0 cohorts are older populations (mean G0 mothers’ age ~56 years [range: 41–75]; mean G0 partners’ age ~61 years [range: 41–89]). Furthermore, the reported associations in the ALSPAC COVID-19 questionnaire data are likely to be specific to the data collection process (i.e. voluntary participation in a long-running birth cohort study), and may not be generalised to studies that acquired COVID-19 data from other sources (e.g. medical records linkage). However, birth cohorts do tend to be more representative of their target population than other study designs, which may minimise potential biases due to selection relative to these other studies (for instance, initial recruitment into ALSPAC included ~75% of the target population ^14^, while UK Biobank only achieved a 5% recruitment rate ^7^). Although we cannot extrapolate findings across cohorts, confidence in any conclusions would be amplified if we found similar results using comparable data from multiple studies/cohorts with different demographics, enrolment processes and data collection strategies (see, for example, work on the impact of COVID-19 on mental health in both ALSPAC and Generation Scotland cohorts ^1^). We therefore encourage other longitudinal studies to perform similar analyses to these, to help researchers plan analyses and interpret their findings.

Nonetheless, we consider that the issues related to selection bias in COVID-19 research described throughout this paper are relevant to all COVID-19 observations studies globally. This is because being infected by SARS-CoV-2, experiencing COVID-19 and the measures that have been used to limit the spread of infection (e.g., lockdowns) will influence who responds to invitations to participate in COVID-19 research. At the same time, who got tested and diagnosed would have been influenced by characteristics such as age, sex, occupation, socioeconomic position and existing co-morbidities, among other factors. However, as the distribution of these characteristics, and the management of the pandemic (e.g., whether universal or selected testing is used) will vary widely between countries, we must be careful in making inferences from our specific results to other populations. Indeed, even within the UK we have shown that many of the characteristics related to response in ALSPAC also do so in UK Biobank, but for several factors the magnitude, and sometimes even the direction, of the associations differed ^42^. The aim of this paper was to highlight key sources of selection bias in COVID-19 research; we feel that most observational studies (including genome-wide association studies and those using genetic data for causal inference, such as Mendelian randomization studies), exploring causes or consequences of COVID-19 are likely to have such biases and thus our conclusions and suggestion that this should be explored in other studies are widely relevant.

It is also possible that rates of response to future COVID-19 questionnaires and associations of candidate predictors may change over time within a cohort like ALSPAC (for instance, because of increased understanding in the importance of COVID-19 research and changes in the restrictions used to manage COVID-19). In our supplementary analyses, we compared candidate predictors of questionnaire invitation and response in the two COVID-19 questionnaires separately. Overall, they appeared similar, but more subtle differences are important to consider when using repeated data across multiple waves of COVID-19 data collection. For instance, among ALSPAC participants who returned the first COVID-19 questionnaire, those who returned the second COVID-19 questionnaire were more likely to be older (i.e., G0 participants), had higher educational qualifications, and had fewer recent financial worries in the first COVID-19 questionnaire ^20^. As we only focused on questionnaires completed early in the pandemic, repeating these analyses with subsequent rounds of COVID-19 questionnaires will be important for researching impacts of the long-term effects of COVID-19 and its management, new variants and on long-COVID.

We also note some specific caveats when interpreting these results and working with these ALSPAC data. First, we could not investigate whether those who reported having had COVID-19 were more (or less) likely to respond to these questionnaires, which may result in selection bias when using this as an exposure or outcome. This risk of bias may be especially concerning as we only have COVID-19 questionnaire data for ~20% of the G1 and G0 mother cohorts (~10% for G0 partners), meaning that COVID-19 status data is missing for ~80% of the cohorts. Additional linkage data – such as from Public Health England COVID-19 testing data – or data from other sources is required to answer such questions ^39^. For instance, recent research using ALSPAC COVID-19 serology test data demonstrates that individuals consenting to have a COVID-19 antibody test were more likely to report having had COVID-19 ^23^. Questionnaire completion may have been similarly biased, thus increasing the risk of selection bias in studies where COVID-19-related outcomes are the outcome of interest.

Second, as ALSPAC data was collected repeatedly using the same questions/instruments, appropriate methods to model this repeated data should be used. Here, for repeated measures we chose a simple method consisting of using the most recent observation, and, if missing, back-filling with previous data. However, if the time-points are not comparable, this may result in bias (e.g., different rates of depression or anxiety at different ages, or different ‘smoking status’ at age 18 vs age 24). Nonetheless, using G1 depression and asthma as examples, we compared different approaches to define these variables (such as using single time-points, forward-filling data and averaging), and found that they were broadly consistent ( *Extended data* ^26^: Table S12; although for depression using data from single time-points gave slightly lower estimates relative to collating over repeated measures). However, we noted that using ‘any history of *X*’ to derive these variables biased cases to those with more data, effectively turning these variables into measures of repeated participation, so we recommend researchers *not* to use this approach. As such, we suggest that researchers apply appropriate statistical techniques when working with these (possibly missing) longitudinal data, such as longitudinal multiple imputation ^43^, latent variable/structural equation modelling (e.g., 44), and/or sensitivity analyses using different variable derivations.

Finally, as 913 G1 children (6% of the whole G1 cohort) were enrolled after the age of seven years, candidate predictors of selection measured during pregnancy and in early childhood will be missing for these children and their G0 parents. This includes potentially important factors associated with selection such as parental education, parental occupational social class, gestational age, maternal parity, maternal age at birth, maternal smoking in pregnancy and parental perinatal depression. Therefore these variables cannot be used to derive weights for these participants if using inverse probability weighting; while if there are few observed auxiliary variables associated with these factors, then multiple imputation will have little information to draw upon when predicting these variables, resulting in greater uncertainty in parameter estimates.

## Conclusion

Missing data from not being sent an invitation or questionnaire and not responding (as well as other sources of missing data) can lead to spurious inferences and counterintuitive results due to selection bias, which may result in incorrect policy recommendations. This is particularly important in the fast-moving area of COVID-19 research. We found several factors associated with selection due to questionnaire invitation and response that may bias findings in COVID-19 research in ALSPAC. This work can be used as a basis for future research using ALSPAC COVID-19 data and highlights the importance of using longitudinal pre-pandemic data to assess potential selection pressures in observational COVID-19 research, and make informed inferences.

## Data availability

### Underlying data

ALSPAC data access is through a system of managed open access. The steps below highlight how to apply for access to the data included in this study and all other ALSPAC data. The datasets presented in this article are linked to ALSPAC project number B3543, please quote this project number during your application. The ALSPAC variable codes highlighted in the dataset descriptions can be used to specify required variables.

1. Please read the ALSPAC access policy which describes the process of accessing the data and samples in detail, and outlines the costs associated with doing so.

2. You may also find it useful to browse our fully searchable research proposals database, which lists all research projects that have been approved since April 2011.

3. Please submit your research proposal for consideration by the ALSPAC Executive Committee. You will receive a response within 10 working days to advise you whether your proposal has been approved.

If you have any questions about accessing data, please email alspac-data@bristol.ac.uk.

Please note that a standard COVID-19 dataset will be made available at no charge (see ^19, 20^); however, costs for required paperwork and any bespoke datasets required additional variables will apply.

### Extended data

Open Science Framework: Questionnaire Invitation/Response and Selection Bias, https://doi.org/10.17605/OSF.IO/TP45V ^26^.

This project contains the following extended data:

- ALSPACSelectionBias_SuppInfo.pdf (supplementary information file: Tables S1–12; Figures S1–26)

### Reporting guidelines

Open Science Framework: STROBE checklist for ‘Bias from questionnaire invitation and response in COVID-19 research: an example using ALSPAC’, https://doi.org/10.17605/OSF.IO/TP45V ^26^.

Data are available under the terms of the Creative Commons Attribution 4.0 International license (CC-BY 4.0).
