# Supplementary material for: Bias from questionnaire invitation and response in COVID-19 research: an example using ALSPAC
Source: Wellcome Open Res. 2022 Jul 8;6:184. Originally published 2021 Jul 19. [Version 2] doi: 10.12688/wellcomeopenres.17041.2 (PMC9294498; doi:10.12688/wellcomeopenres.17041.2)
Supplement: Peer review history

# Peer review: Reviewer response for version 2

**DOI:** 10.21956/wellcomeopenres.19985.r51457

**Published:** 2022-07-18

**Authors:** Dipesh P Gopal

**Affiliations:** 1 Centre of Primary Care, Wolfson Institute of Population Health, Barts and The London School of Medicine and Dentistry, London, UK

Thank you for responding. I have no further comments to make. Thank you. I would be great to see this work indexed.

Is the work clearly and accurately presented and does it cite the current literature?

Yes

If applicable, is the statistical analysis and its interpretation appropriate?

Partly

Are all the source data underlying the results available to ensure full reproducibility?

Yes

Is the study design appropriate and is the work technically sound?

Yes

Are the conclusions drawn adequately supported by the results?

Yes

Are sufficient details of methods and analysis provided to allow replication by others?

Yes

Reviewer Expertise:

Primary Care, Cancer Survivorship, Health Inequalities

I confirm that I have read this submission and believe that I have an appropriate level of expertise to confirm that it is of an acceptable scientific standard.

# Peer review: Reviewer response for version 1

**DOI:** 10.21956/wellcomeopenres.18822.r47518

**Published:** 2022-01-04

**Authors:** Dipesh P Gopal

**Affiliations:** 1 Centre of Primary Care, Wolfson Institute of Population Health, Barts and The London School of Medicine and Dentistry, London, UK

The research is comprehensively undertaken and is a marked improvement on other work using the ALSPAC data by using validated symptom scores where appropriate.

 However, it is unclear what this research adds to the literature especially since previous ALSPAC work showed that people from low socio-economic backgrounds have lower response rates (BMJ Open. 2017 Mar 14;7(3):e014238.). ^1^

 The data presented graphically is difficult to follow and much of the data referenced in the text is in 'Extended data' section. Hugely frustrating for the reader. Could it all be organised so the relevant data is presented?

Is the work clearly and accurately presented and does it cite the current literature?

Yes

If applicable, is the statistical analysis and its interpretation appropriate?

Partly

Are all the source data underlying the results available to ensure full reproducibility?

Yes

Is the study design appropriate and is the work technically sound?

Yes

Are the conclusions drawn adequately supported by the results?

Yes

Are sufficient details of methods and analysis provided to allow replication by others?

Yes

Reviewer Expertise:

Primary Care, Cancer Survivorship, Health Inequalities

I confirm that I have read this submission and believe that I have an appropriate level of expertise to confirm that it is of an acceptable scientific standard, however I have significant reservations, as outlined above.

## References

[1] : Trajectories of urinary incontinence in childhood and bladder and bowel symptoms in adolescence: prospective cohort study. *BMJ Open* .2017;7(3) : 10.1136/bmjopen-2016-014238 10.1136/bmjopen-2016-014238 PMC535329628292756

# Response

**Published:** 2022-07-01

**Authors:** Daniel Smith

**Affiliations:** University of Bristol, UK

We thank the author for their review, and hope that we have addressed any remaining concerns. We also apologise for the length of time taken to respond to the reviewer’s comments; we were expecting a further review before revising the paper, but this never materialised. We have responded to the reviewer’s comments in turn below (with the reviewer's original comments in italics).

*However, it is unclear what this research adds to the literature especially since previous ALSPAC work showed that people from low socio-economic backgrounds have lower response rates (BMJ Open. 2017 Mar 14;7(3):e014238.).1*

 While socioeconomic position (SEP) is indeed a key predictor of response, we also explored other factors which could inform future work using this data. We show that the assessed factors (which are not limited to SEP) predict response to COVID-19 data collections specifically, which may be different from previous ALSPAC data collections. For example, the analysed COVID-19 data was collected through online-only questionnaires (unlike the reference provided) and during a pandemic (which is an additional selection pressure on top of those arising from different data collections and a different time point).

*The data presented graphically is difficult to follow and much of the data referenced in the text is in 'Extended data' section. Hugely frustrating for the reader. Could it all be organised so the relevant data is presented?*

 We understand that the amount of data described may be overwhelming for the reader. Therefore, we tried to be clear giving enough information in the main text to get the overall take-home message. Additional details and further depictions are shown in the extended data for those readers interested in exploring the finer details of this work. We have added a sentence in the ‘statistical analysis’ section of the results (previously just in figure footnotes) to make this clearer:

 “ **Note that, to aid interpretability of the figures displaying these results, we present a range of key candidate predictors of selection in the main text, with additional predictors displayed in the extended data**.”

 We would appreciate more specific feedback on suggestions on how to improve the organisation of the paper; we would be happy to revise the paper accordingly if those suggestions improved clarity.

# Peer review: Reviewer response for version 1

**DOI:** 10.21956/wellcomeopenres.18822.r45126

**Published:** 2021-08-23

**Authors:** Claire Infante-Rivard

**Affiliations:** 1 Department of Epidemiology, Biostatistics and Occupational Health, Faculty of Medicine, McGill University, Montréal, QC, Canada

The authors study selection bias while investigating COVID-19 issues with online questionnaires in the ALSPAC cohorts.

 The results of this descriptive study are important and particularly useful; selection bias, a rather complex and misunderstood bias, is often ignored in the interpretation of results or addressed informally, while potentially present in most studies. The report is very clearly written, the study methods are impeccably presented and thus provide an intelligible model for investigators concerned with the bias but uncertain about how to go about uncovering its presence in their data.

 I have a minor comment about the wording of one aspect of the report. I would strongly suggest changing the wording around the issue of bias related to “being sent a questionnaire”. This is initially interpreted as a meaning bias originating from the research team when selecting who is to be sent a questionnaire whereas it has nothing to do with that. I am sure the authors can come up with a less ambiguous formulation: for example, “subjects who were reachable” or “subjects who could be contacted”, etc.  

 My main comment though is about the need for a more advanced discussion with regards to the effects of selection bias; briefly, the effect can be biased in the sample (and therefore in the target population) or an unbiased in the sample but biased for the target. Without getting into the specific, and sometimes subtle, aspects of these observations, for sure, the reader would benefit from an enhanced discussion on the issue of generalizability from sample results to the target.

 All studies have the goal of interpreting results from the sample to the target population of inferential interest. Few investigators are interested in biomedical results in a few hundred or even a few thousand children or mothers (i.e., the study sample). All are interested in knowing if sample results are generalizable to a well characterized population of children and/or mothers. Whereas the report recognizes that “ALSPAC is not necessarily representative of the general UK population or non-UK population” the target populations at any of the analyses time points in this report remain undefined, with the possible exception of recruitment time, assuming a sampling frame allowed the enumeration of eligible subjects. With the successive follow-ups and the marked losses at each step, defining the target at these steps is a challenge. However, this knowledge impacts on the usefulness of study results, including the ones shown in this report.  

 While the issue of defining the target population of inferential interest may seem theoretical, it becomes very concrete when it comes to generalizing results from a sample meeting the identification conditions. For example, if one were to use IPW or a G-formula approach (which by the way should be added to the list of methods in the report), distribution of key selection-influencing variables from the target would be needed (let’s assume no confounding for simplicity). Should the distribution of these selection-influencing factors be from the eligible at recruitment or from those remaining in the study at later follow-up times? These may well represent different target populations. There are many possible selection models: at entry, at follow-up, at death, for item response. Using IPW for example, should the weights be specific to each mechanism or is the use of a simpler average weight over the mechanisms interpretable? More concretely, from a public health standpoint, assuming generalizations from the sample could be applied, how would the target populations be defined in these applications?

 Another problem arises when the authors use additional children in the G1 cohort; they are defined as eligible but non-participant children: from the ALSPAC literature, it seems impossible to define a sampling frame for these, so the target remains undefined. Generalization with methods previously mentioned could not be performed. Adding these children only makes the results less interpretable with respect to generalization.

 In summary, I think the paper would gain considerable depth if it at least addressed some of the issues raised in the previous paragraphs in the discussion.

 Finally, whereas the authors are prudent about the variables used as driving selection, stating that they are not studying all possible sources of selection bias in ALSPAC, I believe that providing additional insight from the ALSPAC experience could be useful to the readership.  What would a reasonable strategy be to choose the selection-influencing variables for identification (and used with IPW and the G-formula for example). This can be a complex task but most likely best addressed by investigators with an intimate knowledge of the study. Apart from these variables, a reflection on confounders is also needed for identification. Non-causal paths from confounders are familiar but non-causal paths created by selection bias, probably less so. In summary, based on the authors’ profound knowledge of the ALSPAC data and the present study results, what suggestions can they make to investigators with respect to measuring key variables needed for identification and eventually generalization in a cohort study such as ALSPAC.   

Is the work clearly and accurately presented and does it cite the current literature?

Yes

If applicable, is the statistical analysis and its interpretation appropriate?

Yes

Are all the source data underlying the results available to ensure full reproducibility?

Yes

Is the study design appropriate and is the work technically sound?

Yes

Are the conclusions drawn adequately supported by the results?

Yes

Are sufficient details of methods and analysis provided to allow replication by others?

Yes

Reviewer Expertise:

Epidemiology

I confirm that I have read this submission and believe that I have an appropriate level of expertise to confirm that it is of an acceptable scientific standard.

# Response

**Published:** 2022-07-01

**Authors:** Daniel Smith

**Affiliations:** University of Bristol, UK

We are delighted with the positive review and thank the reviewer for their constructive comments. We also apologise for the length of time taken to respond to the reviewer’s comments; we were expecting a further review before revising the paper, but this never materialised. We have responded to the reviewer’s comments in turn below (with the reviewer's original comments in italics).

*I have a minor comment about the wording of one aspect of the report. I would strongly suggest changing the wording around the issue of bias related to “being sent a questionnaire”. This is initially interpreted as a meaning bias originating from the research team when selecting who is to be sent a questionnaire whereas it has nothing to do with that. I am sure the authors can come up with a less ambiguous formulation: for example, “subjects who were reachable” or “subjects who could be contacted”, etc.*

 We appreciate this suggestion and understand the confusion this unusual wording may introduce. However, ‘being sent a questionnaire’ seems our best choice for this outcome for a few reasons. First, it works as a nice counterpoint to the next outcome, ‘returning a questionnaire’. Second, ‘subjects who were reachable’ is not technically accurate, as some participants were reachable but were not sent a questionnaire (if they had declined to participate in questionnaires, for example). As we are comparing participants who were sent vs “not sent” a questionnaire, we feel this is the most appropriate label for this outcome.

 To try and avoid any potential confusion, in the second paragraph of the introduction where we first mention the outcome ‘being sent a questionnaire’, we have expanded the definition to say:

 “The aim of this study was to describe questionnaire invitation and response rates and to explore factors associated with (i) being sent a COVID-19 questionnaire **(i.e., participants who were invited to complete a questionnaire because they had not withdrawn from the study, agreed to participate in questionnaires and had a valid email address)**; {ii) returning a COVID-19 questionnaire…”

*My main comment though is about the need for a more advanced discussion with regards to the effects of selection bias; briefly, the effect can be biased in the sample (and therefore in the target population) or an unbiased in the sample but biased for the target. Without getting into the specific, and sometimes subtle, aspects of these observations, for sure, the reader would benefit from an enhanced discussion on the issue of generalizability from sample results to the target.*

 We appreciate this comment and thank the reviewer for raising this important issue. First, we added a sentence in the first paragraph of the introduction to briefly extend the implications of selection bias:

 “…This can result in selection bias [7– 11], which may be exacerbated by the ways in which data collection has changed during the pandemic, for example, with the increased use of online questionnaires [12, 13]. **This selection bias can lead to incorrect (or biased) estimations of the effect of a risk factor on an outcome, in this case COVID-19 and its related impacts. **Importantly, having an available and rich collection of pre-pandemic data in existing longitudinal cohorts can be useful in efforts to explore potential selection pressures that lead to bias [14– 17].”

**

 In the ‘study design’ section of the methods, we have also included an sentence explicitly defining the target population:

 “ **The target population therefore comprises pregnancies leading to children born in the early 1990s in the former county of Avon, in addition to their mothers and the mothers’ partners. This initial enrolled sample included approximately 75% of the target population and was broadly representative of the wider Avon population (albeit somewhat biased towards mother who were married and from higher socioeconomic backgrounds, and biased away from ethnic minorities);** further details can be found in the published cohort profiles [14, 21, 22]”

 In addition to clearly defining the target population, we have also expanded our discussion with respect to representativeness. While our conclusions regarding selection bias are widely applicable globally for any studies concerned with COVID-19 research, we acknowledge that the patterns of selection are likely to vary for different populations that we would want to make inference to, and have tried to clarify this in the discussion:

**“Nonetheless, we consider that the issues related to selection bias in COVID-19 research described throughout this paper are relevant to all COVID-19 observations studies globally. This is because being infected by SARS-CoV-2, experiencing COVID-19 and the measures that were used to limit the spread of infection (e.g., lockdowns) will influence who responds to invitations to participate in COVID-19 research. At the same time, who got tested and diagnosed would have been influenced by characteristics such as age, sex, occupation, socioeconomic position and existing co-morbidities, among other factors. However, as the distribution of these characteristics, and the management of the pandemic (e.g., universal or selected testing) will vary widely between countries, we must be careful in making inferences from our specific results to other populations. Indeed, even within the UK we have shown that many of the characteristics related to response in ALSPAC also do so in UK Biobank, but for several factors the magnitude, and sometimes even the direction, of the associations differed [https://www.medrxiv.org/content/10.1101/2021.12.10.21267363v1]. The aim of this paper was to highlight key sources of selection bias in COVID-19 research; we feel that most observational studies (including genome-wide association studies and those using genetic data for causal inference such as Mendelian randomization studies) exploring causes or consequences of COVID-19 are likely to have such biases, and thus, our conclusions and suggestion that this should be explored in other studies are widely relevant.”**

*All studies have the goal of interpreting results from the sample to the target population of inferential interest. Few investigators are interested in biomedical results in a few hundred or even a few thousand children or mothers (i.e., the study sample). All are interested in knowing if sample results are generalizable to a well characterized population of children and/or mothers. Whereas the report recognizes that “ALSPAC is not necessarily representative of the general UK population or non-UK population” the target populations at any of the analyses time points in this report remain undefined, with the possible exception of recruitment time, assuming a sampling frame allowed the enumeration of eligible subjects. With the successive follow-ups and the marked losses at each step, defining the target at these steps is a challenge. However, this knowledge impacts on the usefulness of study results, including the ones shown in this report.*

 Thank you for raising this point. As mentioned above, we introduced a definition of the target population (children and associated mothers and their partners born in Avon in the early 1990s), which we believe helps to clarify things. We have now amended the sentence highlighted in the discussion to make it clearer that, as the ALSPAC target population is a relatively small geographically- and temporally-defined area, results may not be generalisable to wider UK and non-UK populations:

 “Additionally, **given the target population (defined above)**, ALSPAC is not necessarily representative of the general UK population or non-UK populations”

 In addition, in the second paragraph of the discussion we have made it clear that, due to selection at each successive stage explored here (sent a questionnaire vs returned a questionnaire vs item non-response), samples using this COVID-19 questionnaire data are not representative of the target population, and therefore at risk of selection bias:

**“Recruitment was intended to be representative of the target population; however, due to loss to follow-up, the analytic samples with COVID-19 questionnaire data are no longer representative of the original ALSPAC study sample (and hence the target population, of which the original ALSPAC sample is broadly representative). **Selection bias may therefore be a potential risk when using ALSPAC COVID-19 questionnaire data and we encourage researchers to carefully consider the results presented here to inform their work. **”**

*While the issue of defining the target population of inferential interest may seem theoretical, it becomes very concrete when it comes to generalizing results from a sample meeting the identification conditions. For example, if one were to use IPW or a G-formula approach (which by the way should be added to the list of methods in the report), distribution of key selection-influencing variables from the target would be needed (let’s assume no confounding for simplicity). Should the distribution of these selection-influencing factors be from the eligible at recruitment or from those remaining in the study at later follow-up times? These may well represent different target populations. There are many possible selection models: at entry, at follow-up, at death, for item response. Using IPW for example, should the weights be specific to each mechanism or is the use of a simpler average weight over the mechanisms interpretable? More concretely, from a public health standpoint, assuming generalizations from the sample could be applied, how would the target populations be defined in these applications?*

 We appreciate this comment following the previous one. In addition to adding g-formula approaches to our list of potential methods, we have given an example of how one could apply inverse probability weights in this study (similar considerations would apply for multiple imputation or g-formula approaches). Given that the enrolled ALSPAC sample includes ~75% of the target population, using this ‘whole sample’ as a proxy for the target population is likely to be a reasonable approximation (even though there are some differences between those enrolled vs non-enrolled, as discussed above), especially given the difficulties in obtaining external information on the non-enrolled pregnancies in the target population:

 "Methods such as multiple imputation, inverse probably weighting, g-formula approaches, simulations, and bounds and parameter searches [11, 30, 34– 38] can be used to help explore and overcome potential selection bias. **For instance, in the case of inverse probability weighting, weights should be derived according to the target population of the study (or using the total enrolled ALSPAC sample as a proxy, if weights derived from the target population cannot be constructed).**”

*Another problem arises when the authors use additional children in the G1 cohort; they are defined as eligible but non-participant children: from the ALSPAC literature, it seems impossible to define a sampling frame for these, so the target remains undefined. Generalization with methods previously mentioned could not be performed. Adding these children only makes the results less interpretable with respect to generalization.*

 We have added the following in the methods section to make clear these children were part of the target population:

 "Since the oldest children were approximately 7 years of age, the study has recruited 913 additional G1 children who did not join originally, but were **part of the original target population** based on date and location of birth."

*In summary, I think the paper would gain considerable depth if it at least addressed some of the issues raised in the previous paragraphs in the discussion.*

 We have added the amendments explained above to make those important points clearer, but we are concerned about the length of this manuscript, particularly the discussion. We hope these modifications improve those previously unclear or misleading issues.

*Finally, whereas the authors are prudent about the variables used as driving selection, stating that they are not studying all possible sources of selection bias in ALSPAC, I believe that providing additional insight from the ALSPAC experience could be useful to the readership.  What would a reasonable strategy be to choose the selection-influencing variables for identification (and used with IPW and the G-formula for example). This can be a complex task but most likely best addressed by investigators with an intimate knowledge of the study. Apart from these variables, a reflection on confounders is also needed for identification. Non-causal paths from confounders are familiar but non-causal paths created by selection bias, probably less so. In summary, based on the authors’ profound knowledge of the ALSPAC data and the present study results, what suggestions can they make to investigators with respect to measuring key variables needed for identification and eventually generalization in a cohort study such as ALSPAC.*

 As we state in the discussion, although we cannot dictate how researchers analyse ALSPAC data, we agree that this might be useful to help guide some readers. In the original version we did present a few suggestions to help researchers (paragraph 6 of the discussion). However, given the length of the discussion already we are hesitant about providing much more additional detail – which could easily fill multiple additional papers! – especially given that we have referenced methods to mitigate selection bias already. Nonetheless, we have introduced a couple of sentences in the discussion to make clear that this study aimed to describe selection pressures in ALSPAC’s self-reported COVID-19 data, and that it is beyond its scope to describe in detail how to mitigate the subsequent bias, but have also provided references to some ALSPAC studies which apply these methods in an effort to overcome potential selection bias:

 " **We have not aimed to mitigate selection bias in this paper, but rather to illustrate how it can be identified.** Methods such as multiple imputation, inverse probably weighting, g-formula approaches, simulations, and bounds and parameter searches [11, 30, 34– 38] can be used to help explore and overcome potential selection bias. […] **For some examples of such methods applied in ALSPAC to mitigate potential selection bias, see [references cited in updated manuscript]. Researchers need to assess the assumptions when using these approaches as **it is not possible to dictate a ‘one-size-fits-all’ approach when working with cohort data such as ALSPAC, as different research questions will be addressed using different variables and methods. **”**

 These references giving examples of how such approaches have been used in ALSPAC to overcome potential selection bias are:

 Cornish RP et al. Factors associated with participation over time in the Avon Longitudinal Study of Parents and Children: a study using linked education and primary care data. Int J Epidemiol. 2021;50(1):293–302. 33057662 10.1093/ije/dyaa192 7938505

 Cornish RP et al. Using linked educational attainment data to reduce bias due to missing outcome data in estimates of the association between the duration of breastfeeding and IQ at 15 years. Int J Epidemiol. 2015;44(3):937–45. 25855709 10.1093/ije/dyv035 4521129

 Lee KJ et al. Framework for the treatment and reporting of missing data in observational studies: The Treatment And Reporting of Missing data in Observational Studies framework. Journal of clinical epidemiology. 2021 Jun 1;134:79-88.

 Cadman T et al. Joint associations of parental personality traits and socio‐economic position with trajectories of offspring depression: Findings from up to 6925 families in a UK birth cohort. JCPP advances. 2021 Oct;1(3):e12028.

 Millard LAC et al. Exploring selection bias in COVID-19 research: Simulations and prospective analyses of two UK cohort studies, MedRxiv. 2021. (https://www.medrxiv.org/content/10.1101/2021.12.10.21267363v1)
